# ESC Congress 2020—Cardiology in the Netherlands

**DOI:** 10.1007/s12471-020-01483-1

**Published:** 2020-08-11

**Authors:** J. J. Piek, A. C. van Rossum

**Affiliations:** 1 Department of Clinical and Experimental Cardiology, Heart Center, Amsterdam Cardiovascular Sciences, Amsterdam UMC, University of Amsterdam, Amsterdam, The Netherlands; 2 Department of Cardiology, Heart Center, Amsterdam Cardiovascular Sciences, Amsterdam UMC, Vrije Universiteit Amsterdam, Amsterdam, The Netherlands

The ESC Congress was organised in Amsterdam for the last time in 2013. Amsterdam, the capital of the Netherlands, was ready to host many thousands of participants for the ESC meeting in 2020. Unfortunately, the event had to be cancelled in its original form due to the outbreak of the COVID-19 pandemic. Nevertheless, the ESC Congress will still take place, although in a virtual form.

Cardiology has a long tradition in the Netherlands, starting with the pioneering work of Willem Einthoven who received the Nobel Prize in 1924 for the development of the string galvanometer to produce an electrocardiogram. Professor Dirk Durrer from Amsterdam was an eminent cardiologist, who is known worldwide for his landmark study on the electrical activation of the human heart, published 50 years ago. Next to his outstanding scientific achievements, he was a visionary man who was able to lead the field of experimental and clinical cardiology. Cardiology became an independent subspecialty of internal medicine because of his dedicated efforts. Moreover, Professor Durrer was the founding father of the Interuniversity Cardiology Institute in the Netherlands, a collaboration between the departments of cardiology of the Dutch university hospitals. Another giant in the Dutch tradition of electrophysiology was Professor Hein Wellens, who unfortunately passed away recently; he gained international recognition for the clinical interpretation and teaching of cardiac arrhythmias.

When considering the great names in cardiology, Professor Paul Hugenholtz deserves a mention for, among many other things, his involvement in the ESC. Back in the early 1970s, he could see the potential and was responsible for major changes in the organisation which are still key features today, such as the annual congresses.

Subsequently, the Netherlands Society of Cardiology was formed and expanded over the years, with now more than 1900 members. The Netherlands Heart Journal is the official peer-reviewed journal of the Netherlands Society of Cardiology. The ESC Congress 2020 provides an excellent opportunity to become acquainted with the Dutch contribution to the broad field of cardiology.

This special issue of the Netherlands Heart Journal, created to mark this year’s congress, covers almost every field of interest in cardiology and is written by internationally recognised Dutch cardiologists.

We hope that the participants of the ESC Congress will enjoy reading the articles on key issues in cardiovascular medicine that illustrate the contribution of Dutch specialists to the field of cardiology and we hope you will enjoy the ESC Congress in its virtual form.

*Jan J. Piek, Editor-in-Chief Netherlands Heart Journal*

*Albert C. van Rossum, Chairman Netherlands Society of Cardiology*

